# Leaf transcriptome analysis of *Medicago ruthenica* revealed its response and adaptive strategy to drought and drought recovery

**DOI:** 10.1186/s12870-022-03918-w

**Published:** 2022-12-02

**Authors:** Rina Wu, Bo Xu, Fengling Shi

**Affiliations:** Key Laboratory of Grassland Resources of the Ministry of Education, College of Grassland Resources and Environment, Inner Mongolia Agricultural University, Hohhot, China

**Keywords:** *Medicago ruthenica*, Drought, Drought recovery, Transcriptome, Adaptive strategy, DEGs

## Abstract

**Background:**

Drought is one of the main causes of losses in forage crop yield and animal production. *Medicago ruthenica* (L.) *cv. Zhilixing* is a high-yielding alfalfa cultivar also known for its high tolerance to drought. We analyzed the transcriptome profile of this cultivar throughout drought stress and recovery and we were able to describe its phased response through the expression profiles of overlapping gene networks and drought-specific genes.

**Results:**

The ABA and auxin signal transduction pathways are overlapping pathways in response to drought and drought recovery in forage crops. *Medicago ruthenica* (L.) *cv. Zhilixing* adopts different strategies at different degrees of drought stress. On the 9th day of drought, transcriptional regulations related to osmoregulation are enhanced mainly through increased activities of carbohydrate and amino acid metabolism, while photosynthetic activities were reduced to slow down growth. With drought prolonging, on the 12th day of drought, the synthesis of proline and other stored organic substances was suppressed in general. After recovery, *Medicago ruthenica* synthesizes flavonoids through the flavonoid biosynthesis pathway to remove accumulated ROS and repair the oxidative damage from water stress. In addition, the regulation of circadian rhythm seems to accelerate the drought recovery process.

**Conclusions:**

*Medicago ruthenica* adapts to drought by regulating the osmoregulatory system and photosynthesis, which appears to involve the ABA and auxin signaling pathways as key regulators. Furthermore, the synthesis of flavonoids and the regulation of the circadian rhythm can accelerate the recovery process. These results enriched our knowledge of molecular responses to drought and drought recovery in *Medicago ruthenica* and provide useful information for the development of new legume forage grass varieties with improved adaptability to drought stress.

**Supplementary Information:**

The online version contains supplementary material available at 10.1186/s12870-022-03918-w.

## Introduction

High salt, freezing conditions, and drought are the major environmental factors that influence plant growth and account for a significant reduction in crop yields [1, 2]. In particular, drought stress is one of the most acute abiotic stresses encountered by plants at a global level [3]. Drought impacts numerous cellular processes, such as signal perception, photosynthesis, and all the molecular and biochemical functions of the cell [4, 5]. It negatively affects plant growth and development by interrupting the processes of cell differentiation, division, and elongation [6], leading to crop yield reduction. Drought limits the utilization of water in plants [3] and plants adapt several strategies to balance optimal water supply to vital organs by (1) closing their stomates to reduce transpiration, (2) increasing their root water uptake, and (3) adjusting internal osmotic processes [7]. In addition, the degree of drought stress gradually increases with time and the response of plants to drought also varies. Therefore, plants adopt distinct adaptation strategies at different stages. Meanwhile, unstable water conditions in fields result in repeated intermittent water deficits, so understanding the resilience of plants after drought is also necessary.

In response to adverse stimuli, plants have evolved a variety of defense mechanisms. Phytohormones are central in the execution of most biological processes, in particular, in response to biotic and abiotic stresses. The extent of water stress triggers specific signaling responses in plants, such as auxin, abscisic acid, brassinosteroids, and ethylene pathways [8–12], and these signaling pathways induce stress-specific transcriptional and physiological responses. Of all plant hormone signaling pathways, ABA is considered a major regulator of abiotic stress. ABA is a signaling molecule that regulates transcription factors involved in stomatal closure that prevent excessive water loss [13–15]. Genetic engineering to improve the function of genes involved in this signal transduction pathway, such as *SnRK2* and *PP2C*, has resulted in the improvement of water use efficiency in plants such as *Arabidopsis thaliana* and wheat [16, 17]. Another important hormone in drought stress is auxin, a regulator traditionally associated with root development. Auxin modulates root architecture under stress [18], which enhances the root’s capacity to absorb water and nutrients [19]. Genes involved in this signal transduction pathway, such as *BoARF6*, *BoARF9* in *Brassica oleracea*, and *AtARF11* in *Arabidopsis thaliana*, can promote lateral root development [20], thereby enhancing water acquisition by roots, finally improving plant performance upon drought. In general, plant hormone signaling pathways play a key role in reducing abiotic stress.

*Medicago ruthenica* is an allogamous, diploid (2n = 16) perennial legume forage. It is widely distributed in alpine and desert grasslands and has the advantages of resistance to cold, drought, salt, and alkali, trampling [21, 22]. Its drought resistance was reported to be better than alfalfa (*Medicago sativa*) and other legume forages. The order is *Medicago ruthenica* (0.52) *> Lotus corniculatus* (0.5) *> Medicago falcata* (0.49) *> Lespedeza bicolor* (0.48) *> Medicago sativa* (0.43) *> Trifolium lupinaster* (0.42) [23]. It has great potential in grassland improvement, ecological management and grass industry development [24, 25]. Importantly, it not only has a high nutritional value (protein content up to 18% during vegetative growth) and good palatability, but also has a higher nutritional utilization efficiency than alfalfa in low-input systems (*Medicago ruthenica* is less affected by nutritional deficits than *Medicago sativa*) [26]. It has no saponin [27]. Excess ingestion of this forage does not cause tympanites in domestic animals. However, its stem is not erect and the forage and seed yields are lower than alfalfa, which limits the widespread adoption and utilization of *Medicago ruthenica*. However, the Zhilixing cultivar grown by Inner Mongolia Agricultural University addressed these undesirable traits. *Medicago ruthenica cv. Zhilixing* is upright and suitable for mowing and cutting. It also has numerous leaves, good forage quality, and high yield. Therefore, in addition to its high forage value and strong resistance, it also has higher forage and seed yield than wild-type *Medicago ruthenica* for cultivation in arid and cold areas [28]. We selected this variety as the experimental material.

Previous studies used *Medicago ruthenica* as parental materials to improve alfalfa tolerance to adverse environments by breeding new alfalfa cultivars [29]. Meanwhile, it can also be a good gene resource to improve the stress resistance of alfalfa and other forage legumes. Studies have shown that the clones *MrDREB1* [30]*, MrLEA2* [31] and *MrDHN3* [32] from *Medicago ruthenica* are related to resistance to abiotic stress in plants. However, the mechanism of resistance of *Medicago ruthenica* to drought and recovery from drought is not yet well understood. Therefore, the present experiment aims to study the changes in *Medicago ruthenica cv. Zhilixing* under drought and drought recovery conditions at the molecular level by RNA-seq. And identified differentially expressed genes under drought and drought recovery by bioinformatics analysis. The analysis also revealed unique and overlapping metabolic processes overrepresented under stress and recovery conditions. These results enriched our knowledge of phased response strategies of *Medicago ruthenica* to drought and drought recovery. Meanwhile, our research put forward new ideas to improve adaptability to abiotic stress in alfalfa and other globally important legume forage grasses.

## Results

### De-novo assembly of transcriptome

In total, 94.35 Gb high-quality reads that ranged from 6.7 to 10.01 Gb per sample were obtained from the transcriptome sequencing of *Medicago ruthenica* leaves. The average call error rates were 0.01% and more than 93% of the bases with very low error rates (< 0.1%). There were 628,999,764 clean reads after removing low-quality sequences (Table 1). A total of 712,926 transcripts were obtained by de novo splicing of data with Trinity. Taking the longest transcripts as unigenes, we obtained 308,449 unigenes with a N50 of 1258 bp. The maximum, minimum, and average lengths were 13,571 bp, 201 bp, and 922 bp, respectively (Table 2). The percentage of unigenes with length of 200–500 bp, 500-1 k bp, 1 k–2 k bp, and longer than 2 k bp was 34.63, 32.91, 23.98, and 8.49%, respectively (Table 3).

**Table 1:** Summary of transcriptome sequencing and assembly results for *Medicago ruthenica cv. Zhilixing*

Sample	Raw Reads	Clean reads	Clean bases	Error(%)	Q20(%)	Q30(%)	GC(%)
A1	55,346,454	53,622,240	8.04G	0.01	98.04	94.74	41.94
A2	69,779,438	66,713,486	10.01G	0.01	97.71	93.85	41.70
A3	53,467,816	51,701,418	7.76G	0.01	98.02	94.70	41.82
B1	54,893,996	52,827,488	7.92G	0.01	97.84	94.40	41.68
B2	50,361,132	48,787,544	7.32G	0.01	98.05	94.78	41.57
B3	47,012,036	44,637,736	6.7G	0.01	97.75	94.28	41.79
C1	57,055,692	54,689,354	8.2G	0.01	97.87	94.45	41.79
C2	46,967,170	45,379,566	6.81G	0.01	97.95	94.70	41.91
C3	46,963,384	45,217,556	6.78G	0.01	97.70	94.20	41.74
D1	58,915,238	56,358,828	8.45G	0.01	97.88	94.50	41.61
D2	56,481,880	54,745,144	8.21G	0.01	98.00	94.64	41.87
D3	56,008,960	54,319,404	8.15G	0.01	98.07	94.80	41.87

A1, A2 and A3 respectively represent 3 biological replicates of the leaf of non-drought stressed *cv. Zhilixing*; B1, B2 and B3 respectively represent 3 biological replicates of the leaf of *cv. Zhilixing* drought-stressed until 9th day; C1, C2 and C3 respectively represent 3 biological replicates of the leaf of *cv. Zhilixing* drought-stressed until 12th day; D1, D2, and D3 respectively represent 3 biological replicates of the leaf of *cv. Zhilixing* on drought recovery after 4 days

**Table 2:** Frequency distribution of splicing length results

	Min length	Mean length	Max Length	N50 Length	N90 Length	Total Nucleotides
Transcripts	201	571	13,571	819	249	406,781,659
Unigenes	201	922	13,571	1258	445	284,452,737

N50/N90: 50%/90% of the assembled bases were incorporated into sequences with length of N50/N90 or longer

**Table 3:** Splicing length distribution

Transcript length interval	200-500 bp	500-1kbp	1 k-2kbp	>2kbp	Total
Number of transcripts	486,365	124,885	75,431	26,245	712,926
Number of unigenes	106,812	101,494	73,961	26,182	308,449

### Sequence annotation

**Fig. 1:**
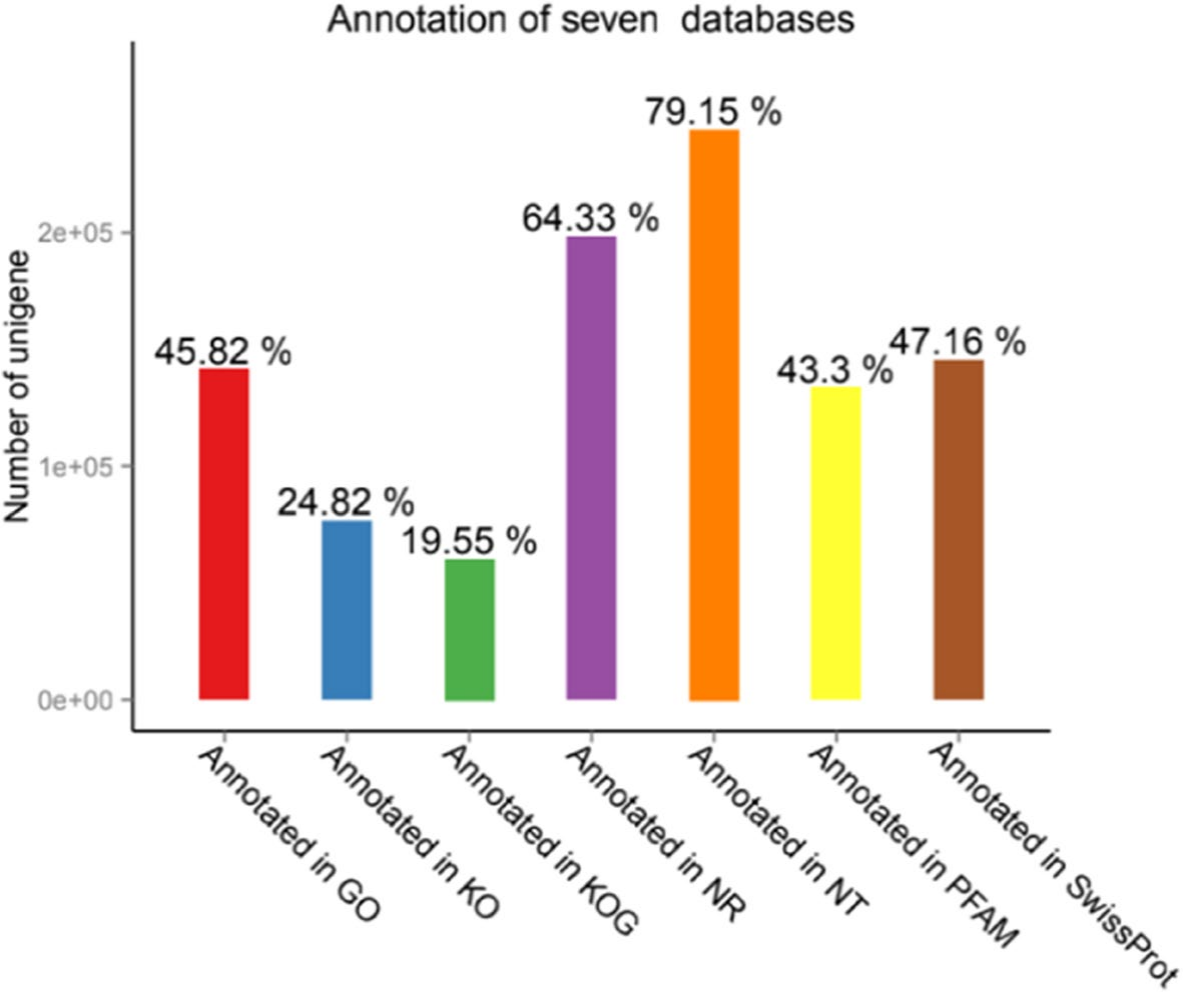
Number of unigenes annotated across seven databases from *Medicago ruthenica* (L.) *cv. Zhilixing* de-novo transcriptome assembly

**Fig. 2:**
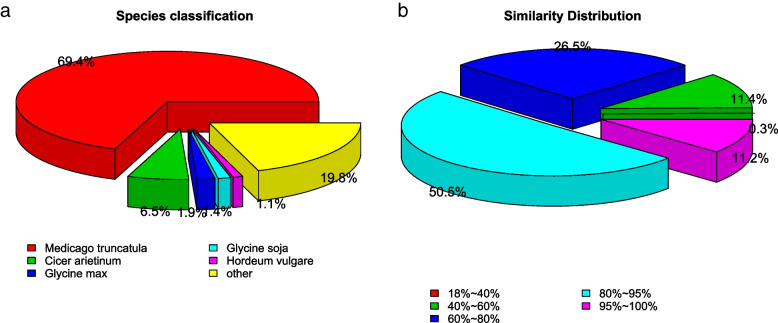
**a** Species distribution of Nr annotation. **b** Similarity distribution of sequence

The 308,449 unigenes showed varying success annotation rates against seven databases: GO (45.82%), KO (24.82%), KOG (19.55%), NR (64.33%), NT (79.15%), PFAM (43.3%) and Swiss Prot (47.16%) (Fig. 1). The number of genes annotated successfully in at least one of the seven databases was 271,580, accounting for 88.04% of the total number of unigenes. And the number of genes annotated in at least two databases was 209,331, accounting for 77.35%. The 61.7% of the sequences were more than 80% similar to the known sequences (Fig. 2b), and nearly 80.2% of the sequences were matched to the following five species sequences: *Medicago truncatula* (69.4%), *Cicer arietinum* (6.5%), *Glycine max* (1.9%), *Glycine soja* (1.4%), and *Hordeum vulgare* (1.1%) (Fig. 2a).

### Identification and analysis of DEGs under drought and drought recovery

**Fig. 3:**
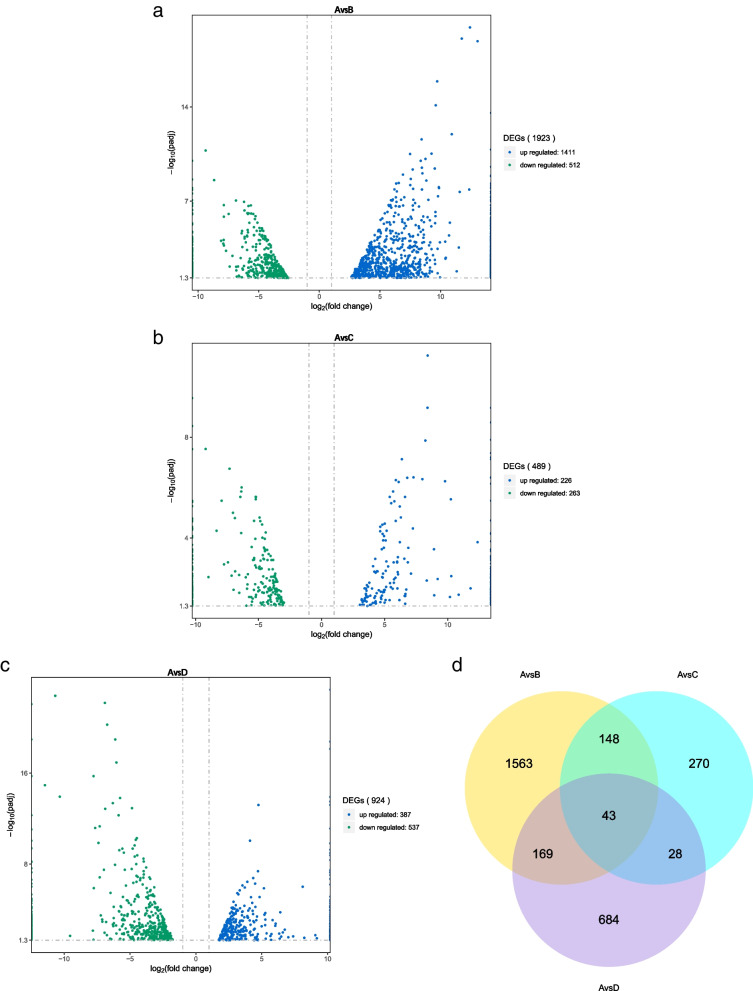
Differential expression analysis of genes. **a**,** b**,** c** Volcano plot of differential expression analysis between different treatments and control. **d** Venn diagram of DEGs among three pairwise differential expression analysis with the control. A = non-drought stressed control, B = drought-stressed until 9th day, C = drought-stressed until 12th day, D = drought recovery after 4 days

Compared to the non-drought challenged control, a total of 2905 unique DEGs were identified across three treatments. At the 9th day drought condition (A vs. B), there were 1923 DEGs (1411 upregulated genes, 512 down-regulated genes), for the 12th day drought condition (A vs. C), 489 DEGs (226 up-regulated genes, 263 down-regulated genes) and for drought recovery condition (A vs. D), 924 DEGs (387 up-regulated genes, 537 down-regulated genes) (Fig. 3). According to Venn analysis, 43 DEGs (8 up-regulated genes, 35 down-regulated genes) on the 9th vs. 12th day of drought vs. 4th day of drought recovery. These genes were not only participate in the response of *Medicago ruthenica* to drought, but also related to the process of drought recovery. These 43 common genes across the three drought treatments set in this study hold promise for enhancing plant drought adaptation (Additional file 1: Table S1; Fig. 3d).

### GO enrichment analysis of DEGs under different treatments

**Fig. 4:**
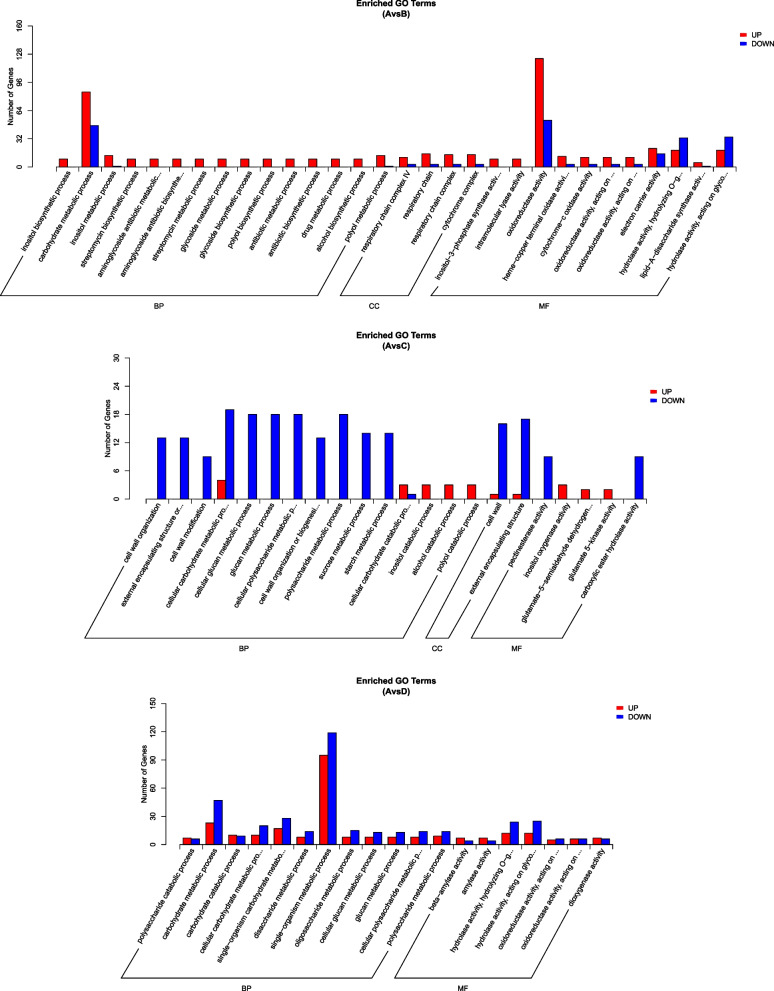
GO classification of DEGs. A = non-drought stressed control, B = drought-stressed until 9th day, C = drought-stressed until 12th day, D = drought recovery after 4 days. A vs. B: GO annotation of DEGs in 9th day drought condition. A vs. C: GO annotation of DEGs in 12th day drought condition. A vs. D: GO annotation of DEGs after drought recovery. BP: biological process, CC: cellular component, MF: molecular function

GO enrichment (FDR ≤ 0.05) showed that 1319 DEGs were annotated successfully in the 9th day drought condition (A vs. B) (987 upregulated genes, 332 down-regulated genes). These DEGs’ molecular functions were associated with the up-regulated activities of oxidoreductase and electron transport, and the down-regulated hydrolase-related genes. The significantly enriched processes were oxidation-reduction (126 up-regulated genes, 48 down-regulated genes), carbohydrate metabolism (85 up-regulated genes, 47 down-regulated genes) and development (126 up-regulated genes, 48 down-regulated genes) (Additional file 2: Table S2; Fig. 4).

In 12th day of drought (A vs. C), 316 DEGs were successfully annotated (132 up-regulated genes, 184 down-regulated genes). Most of the genes were involved in carbohydrate metabolism processes (glucan, sucrose, starch, disaccharide, and oligosaccharide) and were connected with the activities of pectinase, antioxidants, oxidoreductase, and peroxidase. The DEGs were also significantly enriched in cell wall-related biological processes (cell periphery, cell wall organization/modification) and all of them were down-regulated. In the cellular component category, the cell wall and external encapsulating structure were the dominant enriched terms. The up-regulated genes were involved mainly in inositol metabolism, a series of catabolic processes (catabolism of carbohydrates, polyols, inositol, and so on), and were related to enzyme activity (Additional file 3: Table S3; Fig. 4).

Six hundred thirty-two DEGs were annotated successfully in drought recovery condition (A vs. D) (263 up-regulated genes, 369 down-regulated genes). Most of the DEGs involved in carbohydrate (glucan, oligosaccharide, disaccharide, polysaccharide) metabolic processes were down-regulated and related to hydrolase activity. The up-regulated genes mainly participated in catabolism processes (like polysaccharides) and were related to amylase activity (Additional file 4: Table S4; Fig. 4).

### KEGG enrichment analysis of DEGs under different treatment

**Fig. 5:**
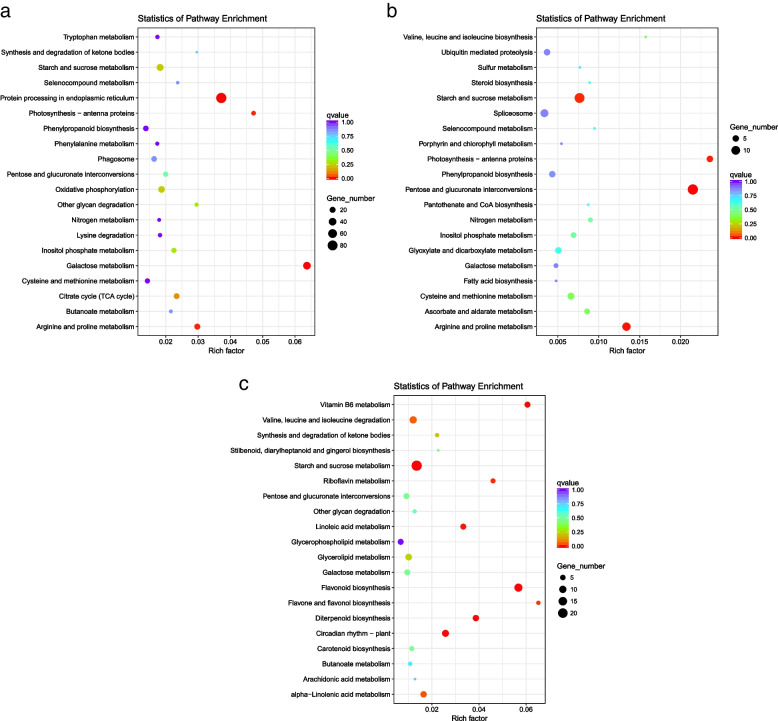
KEGG annotation of DEGs. **a** KEGG annotation of DEGs between control and 9th day drought condition (A vs. B). **b** KEGG annotation of DEGs between control and 12th day drought condition (A vs. C). **c** KEGG annotation of DEGs between control and drought recovery (A vs. D)

KEGG enrichment showed that unigenes were assigned to 104 pathways (*p*-value < 0.05). The enrichment of metabolic pathways differed under different conditions. In 9th day drought condition (A vs. B), most of the DEGs were enriched in galactose metabolism, arginine and proline metabolism, starch and sucrose metabolism, being up-regulated in response to drought. However, the genes enriched in photosynthesis-antenna proteins were mostly down-regulated (Additional file 5: Table S5; Fig. 5a). In 12th day drought condition (A vs. C), most of the DEGs were enriched in arginine and proline metabolism, inositol phosphate metabolism and valine, leucine and isoleucine degradation, all being up-regulated. While those enriched in photosynthesis-antenna proteins, starch and sucrose metabolism were down-regulated (Additional file 6: Table S6; Fig. 5b). During drought recovery condition (A vs. D), most of the DEGs involved in flavonoid biosynthesis, circadian rhythms-plant, vitamin B6 metabolism, and diterpene biosynthesis were up-regulated. While the genes involved in starch and sucrose metabolism, the valine, leucine and isoleucine degradation were down-regulated in recovery process of *Medicago ruthenica* (Additional file 7: Table S7; Fig. 5c). In conclusion, the response mechanism of *Medicago ruthenica* differed in the three phases of drought set in the study.

### Overlapping hormone signal transduction pathways under drought and drought recovery

We analyzed the DEGs involved in plant hormone signal transduction. The results showed that DEGs annotated to seven hormone signaling pathways: auxin, cytokinin, abscisic acid, ethylene, brassinolide, salicylic acid and jasmonic acid. DEGs are mainly involved in auxin, abscisic acid, cytokinin and salicylic acid signaling pathways in 9th day drought condition, auxin and abscisic acid signaling pathways in 12th day drought condition. DEGs are mainly involved in all hormone signaling pathways except cytokinin in drought recovery. Thus, auxin and ABA are overlapping hormone signaling pathways involved in different drought stages and recovery processes (Table.4).

**Table 4:** Overlapping hormone signaling pathways under different treatment

Hormone signal transduction pathway	Treatment		
	A vs. B (9th day drought)	A vs. C (12th day drought)	A vs. D (drought recovery)
IAA (Auxin)	3	1	1
ABA (Abscisic acid)	10	1	1
CTK (Cytokinin)	3	0	0
ETH (Ethylene)	0	0	1
BR (Brassinosteroid)	0	0	1
JA (Jasmonic acid)	0	0	2
SA (Salicylic acid)	1	0	2

### Quantitative real-time PCR analysis

**Fig. 6:**
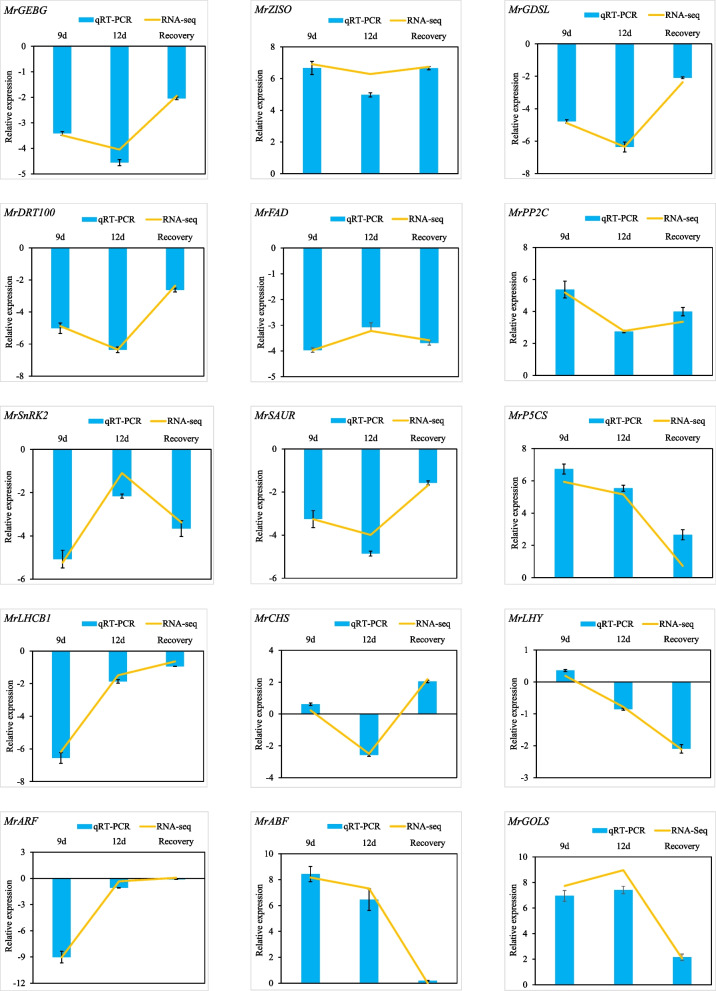
qRT-PCR profiles of some differentially expressed genes at 9th, 12th and drought recovery

In order to verify the accuracy of transcriptome sequencing, 15 DEGs were selected for qRT-PCR, for instance, *MrGEBG* (glucan endo-1,3-beta-glucosidase), photosynthesis related gene carotene isomerase *MrZ-ISO*, a lipid metabolism related gene *GDSL* lipase, DNA damage repair/tolerance protein *MrDRT100*, a fatty acid desaturase *MrFADS*. These genes were randomly selected from the 43 DEGs common to all three treatments. They were mainly involved in carbohydrate and lipid metabolism and carotenoid biosynthesis metabolic processes. *MrGEBG* (~ − 1.9 to − 4.1 folds), *MrGDSL* (~ −2.3 to − 6.3 folds), *MrDRT100* (~ − 2.4 to − 6.3 folds) and *MrFAD* (~ −3.2 to − 4.0 folds) were down-regulated. *MrZ-ISO* (~ 6.3 to 6.9 folds) was up-regulated. Besides, the key genes involved in the metabolic pathways that significant enriched at different stages were also detected, such as *MrPP2C*, *MrP5CS*, *MrABF, MrSnRK2, MrSAUR, MrLHCB1*, *MrARF*, among others. *MrPP2C* (~ 2.8 to 5.2 folds), *MrP5CS* (~ 0.73 to 5.9 folds), *MrABF* (~ 7.3 to 8.2 folds) were up-regulated. *MrSnRK2* (~ − 1.1 to − 5.2 folds)*, MrSAUR* (~ − 1.7 to − 4.0 folds)*, MrLHCB1* (~ − 0.6 to − 6.2 folds) and *MrARF* (~ − 9.0 folds) were down-regulated*.* The expression patterns detected in qRT-PCR fit well with those in the RNA-seq analysis (Fig. 6). The results demonstrated that DEGs identified by transcriptome sequencing were reliable.

## Discussion

In our study, a large number of transcripts of *Medicago ruthenica* were obtained by transcriptome sequencing. Q20 and Q30 of clean reads were above 97 and 93% respectively. The GC content was above 41%. These showed the good sequencing data. Trinity was used for data de-novo splicing, and the N50 length was 1258 bp. This better assembly effect was convenient for subsequent analysis. Through functional annotation, the genes annotated successfully in at least two of the seven databases accounting for 77% of the total number of unigenes. And *Medicago ruthenica* has the highest homology with *Medicago truncatula*. Some genes were selected to verify the accuracy of the transcriptome data. And the qRT-PCR results showed that the genes expression was consistent with the transcriptome data.

### Overlapping genes under both stress and recovery conditions

Our analyses showed that a total of 43 DEGs responded consistently throughout drought and drought recovery. These genes were mainly associated with lipid and carbohydrate metabolism (sucrose, starch, disaccharides, glucan, oligosaccharides, polysaccharides) and carotenoid biosynthesis. These genes could be potential candidates for improving plant stress tolerance.

Z-ISO a key enzyme in the dynamic control of carotenoid biosynthesis, which controls carotenogenesis in a redox-regulated manner through a heme cofactor [33] in facilitating plant adaptation to environmental stress. And Z-ISO expression positively correlated with carotenoid content in tomato [34]. Carotenoids act as photo-protectors, antioxidants, and precursors of phytohormones such as ABA [35]. Its functions are central to plant growth and development. Studies indicating possible involvement of Z-ISO in the evolutionary adaptation of plants to environmental changes [34] and in the breeding of cultivars with consolidated valuable traits. The inhibition of Z-ISO expression blocks the production of carotenoids and end-products of the carotenoid pathway (ABA), resulting in abnormal chloroplast development, reduced chlorophyll levels, and decreased photosynthesis [36–38]. Therefore, it can be a major factor affecting crop yield and be a critical gene for maximizing plant fitness in response to environmental changes [34]. In our study, *MrZ-ISO* involved in carotenoid biosynthesis was up-regulated (~ 6.3 to 6.9 folds) in drought condition, its expression promoted the synthesis of carotenoids followed by synthesis of ABA in plastids [39]. *Medicago ruthenica* used this gene to promote the biosynthesis of ABA which is not only conducive to stomatal closure but also cause leaf senescence and abscission. Besides, *Medicago ruthenica* expresses *MrZ-ISO* to produce antioxidants such as astaxanthin and lutein and thereby eliminate the ROS accumulated under drought conditions.

In addition, we also noticed a down-regulated gene *MrDRT100* (~ − 2.4 to − 6.3 folds) associated with protein binding*.* It belongs to LRR-RLK. As plant signal recognition molecules, leucine repeat rich (LRR) proteins play an important role in signal transduction [40]. The LRR-RLK protein kinase (LRR-RLK) is the largest subclass of plant receptor kinase (RLK) family and plays a key role in plant development and stress responses [41]. For example, *LP2*, a member of the LRR-RLKs family in rice, was down-regulated under drought and ABA stress. The plants over expressing *LP2* had less H_2_O_2_ accumulation and larger leaf stomatal aperture [42]. Up to now, there are few reports on the effect of *DRT100* on drought and drought recovery. Our results showed the role of *MrDRT100* in drought adaptability. It is speculated that the gene can help *Medicago ruthenica* resist stress.

### Overlapping hormone signal transduction pathways under both stress and recovery conditions

Studies showed that plant activates the phytohormone signaling during drought spells [43]. Generally, hormones that mediate stress responses have been characterized under individual stress, but the hormone signal pathways that get activated under both drought and drought recovery remain unclear. ABA and auxin signal pathways were involved in regulating both the response to drought and the recovery process in *Medicago ruthenica*. Therefore, the two signaling pathways may be the key regulators of *Medicago ruthenica* response to drought and drought recovery [44].

ABA not only promotes plant senescence and abscission, but also plays an important role in regulating embryo development, stress and stomatal movement [45]. Stimulated ABA activity is a well-characterized hormone response under drought [46]. Drought changes the soil water potential and stimulates the rapid increase of ABA in plants. Du [47] has found that the ABA metabolism-related genes such as *PYL*, *PP2C* and *SnRK* in plant were responsive to drought. Low expression of *PP2C* slowed down the inhibition of *SnRK* expression, and expression of *SnRK* induced stomatal closure. However, the results of our study were not in accordance with the above. In our study, the expression of the two genes was similar to common vetch (*Vicia sativa* L.) [48], whereby *MrPP2C* was up-regulated, whereas *MrSnRK2* was down-regulated, which promoted the up-regulation of *MrABF1* and induced stomatal closure. In addition, *ABF* belongs to the bZIP transcription factor family can also participate in the response of plants to abiotic stress (high salinity, dehydration, cold, and ABA) [49]. Plants use the expression of *ABF* to suppress rapid water loss and stomata opening [50]. Heterologous expression of *CbABF1* in tobacco improved plant tolerance to freezing and drought [51]. These data suggest that the proper regulation of *MrPP2C*, *MrSnRK2* and *MrABF1* is key to enhancing the adaptability of *Medicago ruthenica* to drought and drought recovery. *Medicago ruthenica* use ABA signaling pathway to close stomatal and promote leaf abscission which limit excessive water loss and save energy to maintain survival.

Root is the main organ for plants to absorb water. Therefore, root morphology regulation under stress is an important aspect of plant adaptation to adversity. In drought condition, root grow toward zones with higher water content to optimize the root system architecture for water acquisition [52] and the process is mediated by auxin signaling [53]. In *Medicago ruthenica*, drought also affected the expression of genes in auxin signal transduction pathway. In our study, *MrARF* was down-regulated in the leaf which repressed the expression of *MrSAUR*. As transcriptional regulators, the *ARFs* directly contribute to auxin signal transposition and are involved in other stress responses [54]. *AcARFs* [55] and *EgARFs* [56] expressed differentially under various abiotic stresses. *SlARF2* [57] and *DnARF11* [58] proved to be involved in root growth. The *SAUR* gene family is the largest part of the early auxin-responsive gene families [59]. It plays an important role in auxin signal response, root development and stress resistance. Study proves that overexpression of *AsSAUR71* in *Agrostis stolonifera* inhibits root growth [60]. *MrSAUR* and *MrARF* participate in the adaptation process of *Medicago ruthenica* to drought and drought recovery. *Medicago ruthenica* maybe use auxin signaling pathway to regulate the root development which increased its nutrients and water absorption. Thereby, *Medicago ruthenica* improves its tolerance to stress.

### Unique adaptive strategies under stress and recovery

In drought-challenged environments, plants usually exhibit a complex network regulation mechanism to change the gene expression level and promote the interaction between biochemical and molecular processes. In this way, the plant could adapt to water deficiency. In addition to the overlapping genes, there were also specific DEGs in different treatment. This finding suggested that *Medicago ruthenica* adopted different strategies to response to different severities of drought and drought recovery. Therefore, we analyzed the GO and KEGG of DEGs in *Medicago ruthenica* under different treatments. Identifying genes and biological processes altered in response to each treatment will advance our understanding of unique adaptation strategies exhibited under drought and recovery.

### Adaptive strategy of *Medicago ruthenica* on the 9th day drought

On the 9th day of drought, *Medicago ruthenica* mainly up-regulated the DEGs in “galactose metabolism”, “arginine and proline metabolism” and down-regulated the DEGs in “photosynthesis-antenna proteins”. *Medicago ruthenica* adopts two major ways to the drought stress. First, it actively adjusts osmotic processes to maintain physiological water balance [7]. In this work, *Medicago ruthenica* simultaneously changed expression of *MrGOLS* in “galactose metabolism” and *MrP5CS* in the “arginine and proline metabolism” to enhance the water holding capacity to presumably improve the drought tolerance. *MrGOLS* (2.1 ~ 9.0 folds) and *MrP5CS* (~ 5.9 folds) was up-regulated during drought. Proline is a major osmo-protectant synthesized in large amounts in arid environment [61, 62]. And galactose, as a soluble sugar, played an important role in osmoregulation. Secondly, *Medicago ruthenica* seem to adapt to drought stress by slowing down its growth. Our study revealed that photosynthesis was inhibited under drought. *MrLHCB1* identified in “photosynthesis-antenna proteins” were down-regulated (~ − 6.2 folds) and this result was similar to those of De [63], Hanf [64] and Behringer [65]. *LHCB*1 mainly involved in light harvesting. Consequently, the photosynthetic activity of *Medicago ruthenica* was suppressed likely due to the decrease of light harvesting capacity.

### Adaptive strategy of *Medicago ruthenica* on the 12th day drought

On the 12th day of drought, down-regulated DEGs mainly involved in the carbohydrate metabolism (starch and sucrose) and photosynthesis (photosynthesis-antenna proteins) of *Medicago ruthenica*, whereas the up-regulated DEGs were related to amino acid metabolism and catabolic processes of organic substances. *Medicago ruthenica* inhibited the carbohydrate metabolism and photosynthesis to reducing the nutrient and energy consumption for a further step. The amino acid metabolism was promoted. The *MrP5CS* in “arginine and proline metabolism” was still significantly up-regulated*. Medicago ruthenica* continued to synthesize proline to relieve osmotic pressure and resist drought. In order to maintain energy supply, a series of catabolic processes (including catabolism of carbohydrates, polyols, inositol, alcohols and organic hydroxyl compounds) were activated under severe drought. This finally diminished the accumulation of biomass. The forage legume mainly relied on the synthesis of proline and the decomposition of other stored organic substances to carry out osmotic adjustment, obtain energy and sacrifice part of biomass to maintain the metabolism. This was a passive response to severe drought.

### Adaptive strategy of *Medicago ruthenica* in 4th day drought recovery

*Medicago ruthenica* up-regulated most of the genes enriched in “flavonoid biosynthesis” and “plant circadian rhythm” 4th day into the drought recovery process. *CHS* is a key gene in flavonoid biosynthesis, and it’s up-regulation promoted the synthesis of flavonoids [66]. This favors the repair of oxidative damage under severe drought. Moreover, *MrCHS* (~ 2.2 folds) was also involved in “plant circadian rhythm”, and it’s up-regulation might be beneficial to UV-B protection of *Medicago ruthenica* leaves. In addition, “plant circadian rhythm” was also remarkably enriched in the recovery process. Studies had confirmed that some critical drought genes and physiological activities induced by drought showed diurnal response pattern [67]. Hence, the circadian rhythms were directly related to the response of plants to drought [68]. However, in our study, the plant circadian rhythm was more significantly enriched in recovery process than water deficit. In additional, the down-regulated gene *MrLHY* (~ − 2.1 folds) in this pathway might be involved in the light signal network transmission. The *GmLHY* negatively controlled drought tolerance of soybean [69]. *Medicago ruthenica* might accelerate the recovery process by regulating the circadian rhythm.

## Conclusion

This study found the overlapping signal transduction pathways (ABA and IAA) and DEGs of *Medicago ruthenica* in response to drought and drought recovery. In addition, we also uncovered unique metabolic processes. On this basis, we did a preliminary analysis about the phased response and adaptive strategy of the plant to different degrees of drought and drought recovery.

In 9th day drought condition, *Medicago ruthenica* improved the osmoregulation ability to cope with drought by promoting carbohydrate and amino acid metabolism. *Medicago ruthenica* also inhibited photosynthesis to slow down the growth. In 12th day drought condition, *Medicago ruthenica* reduced the material and energy consumption by inhibiting the carbohydrates metabolism and photosynthesis. Meanwhile, *Medicago ruthenica* also relied on the synthesis of proline and the decomposition of other stored organic substances to carry out osmoregulation, obtain energy and sacrifice part of biomass to maintain its metabolic homeostasis. During drought recovery process, *Medicago ruthenica* synthesized flavonoids to remove the accumulated ROS and repair the oxidative damage under water stress. In addition, *Medicago ruthenica* regulated circadian rhythm to accelerate the recovery process. These results provide a reference for the later study of the phased response strategy of plants to drought and drought recovery*.* In addition, the study enriches the promising candidate drought genes. This is useful information for the development of varieties with improved adaptability to abiotic stress in the globally important legume forage grass like alfalfa and other forage crops.

## Methods

### Sample collection and preparation

*Medicago ruthenica* (L.) *cv. Zhilixing* seeds harvested at the experimental plot in Inner Mongolia Agricultural University. It is located in Hohhot (111°41′ E, 40°48′ N), Inner Mongolia, China. Material preparation method please refer to previously described [70]. The experiment was divided in two groups: the control group which received normal watering. And the treatment group was not watered for 12 days when the leaves of seedlings wilted, turned yellow and fell off, from which point watering was administered to sufficient moisture for several days. After four days watering, leaf samples were then sampled. Thus, there were 3 treatments: the 9th, 12th day of the drought, and 4th day after recovery according to the preliminary experiment [71]. The fourth or fifth mature leaf was sampled from three plants (biological replicates) from each treatment conditions. In the control group, leaf samples were likewise collected but only once, on the 9th day of normal watering. In all, 12 leaf samples were collected which were then immediately frozen in liquid nitrogen and stored at − 80 °C for RNA extraction. All materials are stored in the Key Laboratory of Grassland Resources of the Ministry of Education in Inner Mongolia Agricultural University (voucher ID: BXDzhilixing).

### RNA extraction and transcriptome sequencing

Total RNA was extracted using a TRIzol reagent (Invitrogen, Carlsbad, CA, USA), following the manufacturer’s protocol. RNA purity was checked using a NanoPhotometer® spectrophotometer (IMPLEN, CA, USA). RNA concentration was measured using a Qubit® RNA Assay Kit in a Qubit® 2.0 Fluorometer (Life Technologies, CA, USA). RNA integrity was assessed using a RNA Nano 6000 Assay Kit of an Agilent Bioanalyzer 2100 system (Agilent Technologies, CA, USA).

Sequencing libraries were generated using a NEBNext® Ultra™ RNA Library Prep Kit for Illumina®(NEB, USA). The library fragments were purified with an AMPure XP system (Beckman Coulter, Beverly, USA) to select cDNA fragments of 250 ~ 300 bp in length. Then, 3 μL of USER Enzyme (NEB, USA) was used with size-selected, adaptor-ligated cDNA at 37 °C for 15 min followed by 5 min at 95 °C before PCR. Then, to perform PCR with Phusion High-Fidelity DNA polymerase, we used the universal PCR primers and the index (X) primer. Finally, the PCR products were purified (AMPure XP system), and the library quality was assessed (Agilent Bioanalyzer 2100 system).

The clustering of the index-coded samples was performed on a cBot Cluster Generation System using a TruSeq PE Cluster Kit v3-cBot-HS (Illumina) according to the manufacturer’s instructions. After cluster generation, the library preparations were sequenced on an Illumina NovaSeq 6000 platform and paired-end reads were generated.

Raw data (raw reads) of fastq format were firstly processed through in-house perl scripts. In this step, clean data (clean reads) were obtained by removing reads containing adapter, reads containing ploy-N and low quality reads from raw data. At the same time, Q20, Q30, GC-content and sequence duplication level of the clean data were calculated. All the downstream analyses were based on clean data with high quality. Transcriptome assembly was accomplished based on the left.fq and right.fq using Trinity [72] with min_kmer_cov set to 2 by default. All other parameters also set default.

### Unigene annotation

Gene function was annotated based on the following databases: NR (NCBI non-redundant protein sequences) (http://www.ncbi.nlm.nih.gov), NT (NCBI non-redundant nucleotide sequences) (http://www.ncbi.nlm.nih.gov), Pfam (Protein family) (http://pfam.sanger.ac.uk/), KOG (euKaryotic Ortholog Groups) (http://www.genome.jp/kegg/ko.html), Swiss-Prot (A manually annotated and reviewed protein sequence database) (http://www.ebi.ac.uk/uniprot/), KO (KEGG Ontology) (https://www.genome.jp/kegg/ko.html), and GO (Gene Ontology) (http://www.geneontology.org/). Reference gene source used was *Medicago truncatula*.

### Analysis of DEGs

The differential gene expression analysis was performed using the DESeq2. Genes with an adjusted *P*-value < 0.05 and |log2FC| ≥ 1 (the expression difference was more than twice) were identified as DEGs. Bioinformatic analyses of DEGs were carried out on the Novogene Bio-Cloud Platform. The GO enrichment analysis of DEGs was implemented by the GOseq R packages based on the Wallenius non-central hyper-geometric distribution [73]. KEGG [74] was used for understanding high-level functions of the biological system based on molecular-level information (http://www.genome.jp/kegg/). We used KOBAS [75] software to test the statistical enrichment of DEGs in the KEGG pathways.

### Real-time quantitative PCR analysis

The qRT-PCR analysis was performed to validate the DEGs identified by transcriptome sequencing. Total RNA was extracted as described before. An UEIris II RT-PCR System was used for first-strand cDNA Synthesis (with dsDNase) (US Everbright, Suzhou, China). The PCR mixture contained 4 μL of UEIris II RT MasterMix (5X), 1 μL of dsDNase, 14 μL of RNase-free water, and 1 μg of mRNA template. The program was set at 37 °C for 2 min (removing genomic DNA from total RNAs) then 55 °C for 10 min, followed by 85 °C for 10 s. The qRT-PCR assays were performed using a Fast Super EvaGreen® qPCR Master Mix (US Everbright, Suzhou, China) in an ABI 7500 system (Applied Biosystems, USA) with the following program: 95 °C for 2 min; 45 cycles of 95 °C for 10 s, 55 °C for 10 s, 72 °C for 30 s, 95 °C for 15 s, and 60 °C for 60 s. The *MrActin* was selected as the endogenous reference. All the primers were synthesized by Invitrogen (Beijing, China) (Additional file 8: Table S8). A 20 μL of reaction mix contained 10 μL of 2 × Fast Super EvaGreen® Master Mix, 1 μL of 10 × ROX, 5.5 μL of ddH_2_O, 3 μL of cDNA, and 0.5 μg of each primer. The relative expression changes of the endogenous reference and the tested genes were analyzed by the 2^- △△ CT^ method [76].

## Supplementary Information

**Additional file 1: Table S1.** DEGs in AB vs. AC vs. AD.

**Additional file 2: Table S2.** GO functional annotations and the number of DEGs statistics in AB.

**Additional file 3: Table S3.** GO functional annotations and the number of DEGs statistics in AC.

**Additional file 4: Table S4.** GO functional annotations and the number of DEGs statistics in AD.

**Additional file 5: Table S5.** Top 20 KEGG pathways of DEGs in drought AB.

**Additional file 6: Table S6.** Top 20 KEGG pathways of DEGs in drought AC.

**Additional file 7: Table S7.** Top 20 KEGG pathways of DEGs in AD.

**Additional file 8: Table S8.** Primers for qRT-PCR.

## Data Availability

Datasets are available at NCBI project PRJNA655825. The transcriptome datasets are available in the NCBI Short Read Archive (SRA) database with the accession number: SAMN15756116, SAMN15756117, SAMN15756118, SAMN15756119, SAMN15756120, SAMN15756121, SAMN15756122, SAMN15756123, SAMN15756124, SAMN15756125, SAMN15756126, SAMN15756127.
