# Supplementary material for: Leaf transcriptome analysis of Medicago ruthenica revealed its response and adaptive strategy to drought and drought recovery
Source: BMC Plant Biol. 2022 Dec 2;22:562. doi: 10.1186/s12870-022-03918-w (PMC9716755; doi:10.1186/s12870-022-03918-w)
Supplement: Supplementary file 8 — Additional file 8: Table S8. Primers for qRT-PCR. [file 12870_2022_3918_MOESM8_ESM.docx]

Table.S9 Primers for qRT-PCR

| Gene | Primer (5’ to 3’) | |
| --- | --- | --- |
| *MrGEBG* | F:TCCCCTTGAACCATCCCTAACT | R:AACGGCTATTGACATGACACTGAT |
| *MrZISO* | F: CAGATAGTCGTGAGGTGGTAATGTT | R: AGAGCCAAAGGTAGCGATGTG |
| *MrGDSL* | F: ACAGACGCAGCAGTGATAGT | R: GCAGAGGTGGAAGAGCATTATTG |
| *MrDRT100* | F: AGTCCTTTACAGCATCAATGAACAG | R: GTTGAATCCACCACAGAGACCT |
| *MrFAD* | F: TGTGTCGGTGGTCCTGTGTT | R: CGGGTTATGAGTTGGCGAATGG |
| *MrPP2C* | F:CGCTGTTGTTGCCGTTGT | R:CTCGTCACTCCTCTCCGTTAT |
| *MrSnRK2* | F:AAGAAGGAATACGATGGCAAGA | R:GAGATCAGATGACGGCACTC |
| *MrSAUR* | F:GGCTATCTCGCAGTCTATGTTG | R:AGGAATTGTGAGACCACCCATA |
| *MrP5CS* | F:TGCTGCTGTATTCCACAATGC | R:TCAACAAGCCCTCAACTCCT |
| *MrLHCB1* | F:GCAGAGTTGAAGGTGAAAGAAC | R:AGCAAGGTGGTCAGCAAGA |
| *MrCHS* | F:CTGCTCTTATTGTTGGCTCTG | R:TCAATGGCTCCTTCACTATCAG |
| *MrLHY* | F:ACGATGGACAGAAGACGAACA | R:GCGAGGATAAGGATTGCTTGG |
| *MrARF* | F:CCTCTGCCTGAACCTCCAA | R:ATGCGTCACCAGCCACTAA |
| *MrABF* | F:CTTCAACAGCAGCATCAACAACAG | R:ACCATACCAATCCCTCCACCTT |
| *MrGOLS* | F:GGACGGAGACATTCAAGTT | R:GCCACAACATAGCAAGAAC |
| Actin | F: TTGCTGGTCGTGATCTAACTGA | R: TCTGCTGAGGTGGTGAACAT |
